# SARS‐CoV‐2 Omicron variant: Why global communities should take it seriously?

**DOI:** 10.1002/iid3.618

**Published:** 2022-04-19

**Authors:** Shayan Rahmani, Nima Rezaei

**Affiliations:** ^1^ Department of Medical Virology Student Research Committee, School of Medicine, Shahid Beheshti University of Medical Sciences Tehran Iran; ^2^ Division of Medical Research Network of Immunity in Infection, Malignancy and Autoimmunity (NIIMA), Universal Scientific Education and Research Network (USERN) Tehran Iran; ^3^ Division of Immunology Research Center for Immunodeficiencies, Children's Medical Center, Tehran University of Medical Sciences Tehran Iran; ^4^ Department of Immunology School of Medicine, Tehran University of Medical Sciences Tehran Iran

## Abstract

**Introduction:**

In November 26th, 2021 a new strain of SARS‐CoV‐2 was designated by the World Health Organization as a variant of concern and named Omicron. The news broadcasted a global wave of panic and anxiety while many, like 2 years ago, were making themselves ready for the holiday season. After almost a month of its designation, countries from all 6 continents have been reported Omicron from their genomic sequences. This triggered an international alarm about a new era in the Covid‐19 pandemic, where despite the vast amount of vaccinations, a surge in new cases and hospitalizations are reported from all over the world.

**Methods:**

Scientific literature published from November 26, 2021 to March 21, 2022 have been searched and retrieved by using “SARS‐COV‐2”, “Omicron”, “B.1.1.529”, “Covid‐19”, and “global community” keywords from “PubMed”, "Web o “Google Scholar”, and “MedRxiv” databases.

**Results:**

Omicron have been evolved to spread faster than previous variants of concern, but it infects people lesser than other variants, Delta for example. Omicron can also escape vaccine‐induced immunity more than previous SARS‐CoV‐2 variants.

**Discussion:**

Despite possible lower lethal risks than previous strains, Omicron may provide populations with a higher community transmission and a higher hospitalization load, which potentially overwhelm already exhausted health care systems. Therefore, we need to get used to the “New Normal” and maintain health recommendations to help decrease spreading of the virus and buy more time for the scientists to dive deeper into potential ways of tackling Covid‐19, more than ever.

It was on November 26th, 2021 when a new strain of SARS‐CoV‐2, B.1.1.529, was designated by the World Health Organization as a variant of concern and named Omicron.^1^ The news broadcasted a global wave of panic and anxiety while many, like 2 years ago, were making themselves ready for the holiday season. United States, Canada, and the European Union imposed travel restrictions on several Southern African nations amongst fear over the Omicron while countries like Japan closed their borders to non‐citizens for several weeks. Following the global actions against the spreading of the virus, many scientists around the world have been working nonstop to acknowledge how much infectious and immune escape Omicron can be as the rate of new Covid‐19 cases has been rising astronomically. About 4 months of its designation, the United Kingdom with more than 862,000 confirmed cases has the highest amount of Omicron infection globally,^2^ while based on the data from the Centers for Disease Control and Prevention, as of January 8th, Omicron is accounted for almost 98% of Covid‐19 cases in the United States.^3^ This triggered an international alarm about a new era in the Covid‐19 pandemic, where despite the vast amount of vaccinations, a surge in new cases and hospitalizations reported from all over the world.^4^

**Figure 1:**
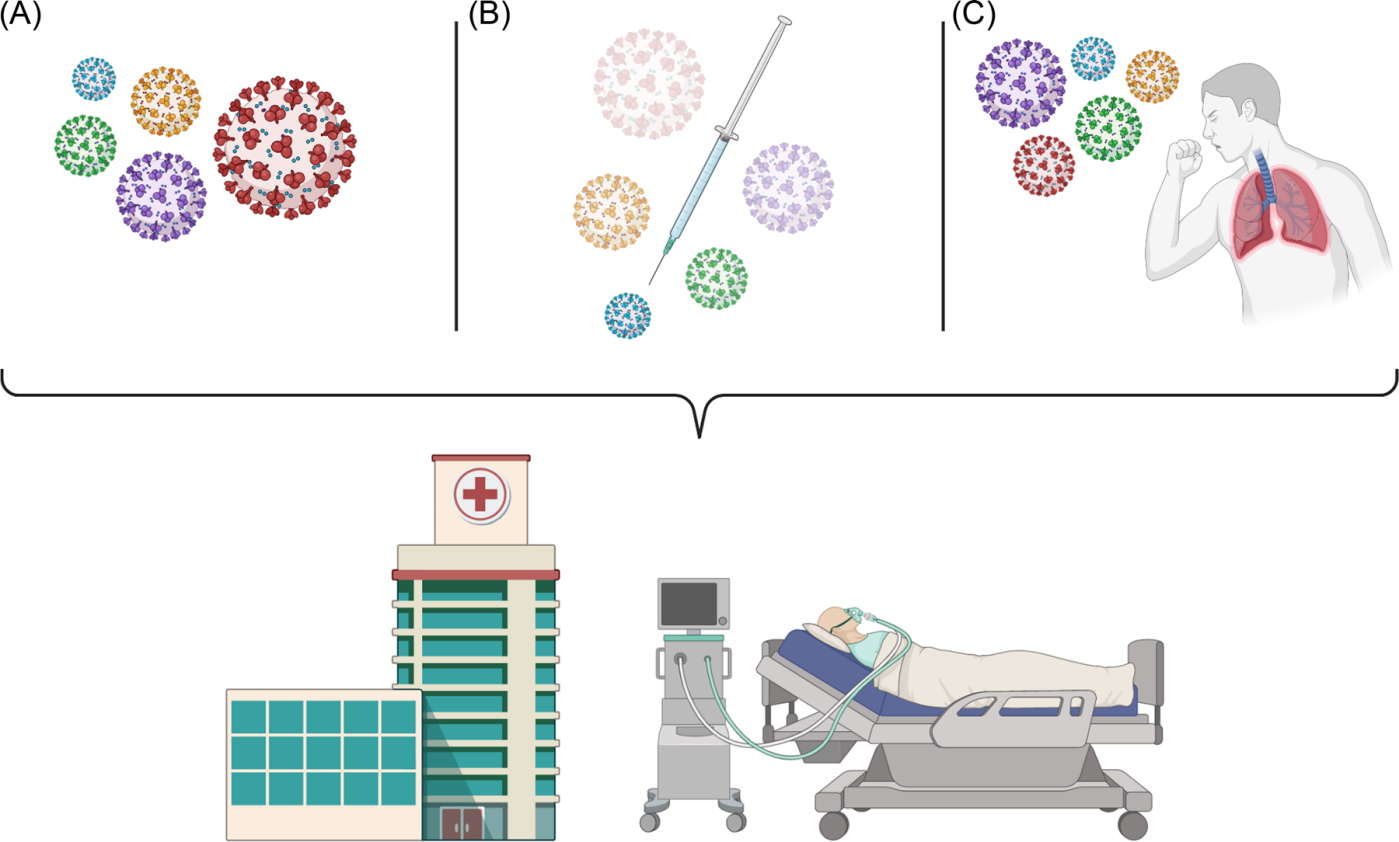
Schematic representation of A) transmissibility B) immune evasion C) Infectivity of several SARS‐CoV‐2 variants of concern. Omicron (red) is more transmissible and immune‐escape than previous variants; however, it is not as infectious as other strains, Delta (purple) for example. Despite lower lethal risks than previous variants, Omicron has a higher community transmission that lead to a higher hospitalization load, which can potentially overwhelm already exhausted healthcare systems

To date, there is less known about how dangerous Omicron can be; However, initial data suggest it spreads faster than previous variants, as it carries the hugest amount of mutations on the Spike protein compared to the other variants of concern.^5^ Besides, it escapes more easily from the immune system as serum samples from both vaccinated and convalescent people neutralize Omicron much lesser than other variants.^6^ On the other hand, the results of other investigations proposed that Omicron infects lung tissues slower and may have a reduced risk of severe infection or death compared to the Delta variant.^7^, ^8^ Therefore, despite possible lower lethal risks than previous strains, Omicron may provide populations with a higher community transmission, and consequently, a higher hospitalization load in the following months, which potentially overwhelm already exhausted health care systems across the world.(Figure 1)^9^ Besides, it has emerged during the meaning time of vaccination of many nations. Although many African nations did not properly vaccinate their people that made them more vulnerable to the chronic viral infection and further mutations of the virus, most of the developed countries vaccinated a greater proportion of their population. However, despite the previous variants that does not impede vaccine‐induced immunity, most of the developed countries have been reporting Omicron from their genomic sequences continuously, a possible threat for the vaccines' effectiveness.^2^, ^10^ This can add to an emerging picture that Omicron may be inherently more transmissible, immune escape, and infectious, even in fully vaccinated or previously infected people, which should put global communities in an alerting situation. Figure 1. Schematic representation of A) transmissibility B) immune evasion C) Infectivity of several SARS‐CoV‐2 variants of concern. Omicron (red) is more transmissible and immune‐escape than previous variants; however, it is not as infectious as other strains, Delta (purple) for example. Despite lower lethal risks than previous variants, Omicron has a higher community transmission that lead to a higher hospitalization load, which can potentially overwhelm already exhausted healthcare systems.

Just like 2 years ago, the original SARS‐CoV‐2 emerged in late 2019 when people were not properly informed, and more importantly, global communities were not ready for such a wave of the disease. After months of investigation on Covid‐19 and its pathophysiology, vaccines and other viral blocking methods such as monoclonal antibodies (mABs) have presented. However, limited clinical benefit of using mABs, several side effects by using vaccines, and obstacles in the treatment of Long Covid‐19 syndrome necessitates the development of efficient clinical methods that may effectively prevent viral entry and IL‐1 blockade at the first place.^11^, ^12^ Meanwhile, maintaining health recommendations such as wearing a well‐fitting mask, washing hands regularly, constant ventilation of indoor spaces, keeping social distancing, testing frequently, and vaccinating along with booster doses still remained essential. On the other hand, global communities should take Omicron even more seriously than before. Improving health care capacities with adequate health care staff, oxygen mask availability, and sufficient intensive care unit beds, along with providing people with precise and swift information about the Covid‐19 is critical as we have entered 2022 and have passed the holiday season.^13^ In the meantime, through the lessons we have learned, it is crucial to provide communities with suitable communication facilitates to protect people from potential social isolation and further mental health problems while breaking the chain of the Covid‐19 infection.^14^, ^15^

It is too hard for us not to make warm human contacts after 2 years of fear, panic, and social anxiety, and too harsh for international affairs not to provide their communities with appropriate services and businesses. As more challenging it might be, we need to get used to the “New Normal” to help decrease the viral spread and buy more time for the scientists to dive deeper into potential ways to tackle Covid‐19 more than ever. Throughout history, nothing is more influential than human powers, and to collaborate internationally, or in other words, to be “Unified” is the essential key to ending the pandemic.

## AUTHOR CONTRIBUTIONS

**Shayan Rahmani**: conceptualized the title, collected data, and prepared the first draft of the manuscript. **Nima Rezaei**: critically revised the manuscript, edited and finalized the draft, and supervised the study. All authors have read and approved the final draft of the manuscript.

## CONFLICTS OF INTEREST

The authors declare no conflicts of interest.
